# Climate research priorities for policy-makers, practitioners, and scientists in Georgia, USA

**DOI:** 10.1007/s00267-018-1051-4

**Published:** 2018-05-23

**Authors:** Murray A. Rudd, Althea F. P. Moore, Daniel Rochberg, Lisa Bianchi-Fossati, Marilyn A. Brown, David D’Onofrio, Carrie A. Furman, Jairo Garcia, Ben Jordan, Jennifer Kline, L. Mark Risse, Patricia L. Yager, Jessica Abbinett, Merryl Alber, Jesse E. Bell, Cyrus Bhedwar, Kim M. Cobb, Juliet Cohen, Matt Cox, Myriam Dormer, Nyasha Dunkley, Heather Farley, Jill Gambill, Mindy Goldstein, Garry Harris, Melissa Hopkinson, Jean-Ann James, Susan Kidd, Pam Knox, Yang Liu, Daniel C. Matisoff, Michael D. Meyer, Jamie D. Mitchem, Katherine Moore, Aspen J. Ono, Jon Philipsborn, Kerrie M. Sendall, Fatemeh Shafiei, Marshall Shepherd, Julia Teebken, Ashby N. Worley

**Affiliations:** 1 Department of Environmental Sciences, Emory University, Atlanta, GA 30322 USA; 2 Department of Environmental Health, Rollins School of Public Health, Emory University, 1518 Clifton Rd NE, Atlanta, GA 30322 USA; 3 Southface Energy Institute, 241 Pine St. NE, Atlanta, GA 30308 USA; 4 School of Public Policy, Georgia Institute of Technology, Athens, GA USA; 5 Atlanta Regional Commission, 229 Peachtree Street NE, Atlanta, GA 30303 USA; 6 Department of Crop and Soil Sciences University of Georgia, Athens, GA 30602 USA; 7 City of Atlanta Office of Resilience, 55 Trinity Av. SW, Atlanta, GA 30303 USA; 8 Georgia Department of Natural Resources Coastal Resources Division, Brunswick, GA USA; 9 University of Georgia Marine Extension and Georgia Sea Grant, The University of Georgia, Athens, GA 30602 USA; 10 Department of Marine Sciences, University of Georgia, Athens, GA 30602 USA; 11 North Carolina Institute for Climate Studies, North Carolina State University, Asheville, NC 28801 USA; 12 Southeast Energy Efficiency Alliance, Atlanta, GA USA; 13 School of Earth and Atmospheric Sciences, Georgia Institute of Technology, 311 Ferst Dr., Atlanta, GA 30332 USA; 14 Chattahoochee Riverkeeper, Atlanta, GA 30306 USA; 15 The Greenlink Group, 695 Pylant St NE, Atlanta, GA 30306 USA; 16 The Nature Conservancy in Georgia, 100 Peachtree St. NW, Suite 2250, Atlanta, Georgia 30303 USA; 17 Georgia Department of Natural Resources Environmental Protection Division, 4244 International Parkway, Atlanta, GA 30354 USA; 18 The College of Coastal Georgia, School of Business and Public Management, One College Drive, Brunswick, GA 31520 USA; 19 Emory University School of Law, 1301 Clifton Road, Atlanta, GA 30322 USA; 20 Center for Sustainable Communities, 100 Flatshoals Ave SE, Atlanta, GA 30316 USA; 21 Institute for Environmental & Spatial Analysis, University of North Georgia, Oakwood, GA 30566 USA; 22 Turner Foundation, Atlanta, GA USA; 23 Center for Sustainability, Agnes Scott College, 141 E. College Ave., Decatur, GA 30030 USA; 24 WSP USA Inc., 845 Spring Street, Unit 204, Atlanta, GA 30308 USA; 25 Sustainable Growth Program, Georgia Conservancy 230 Peachtree Street Suite 1250, Atlanta, GA 30303 USA; 26 AECOM Technical Services, Inc, Atlanta, GA USA; 27 Department of Biology, Georgia Southern University, Statesboro, GA 30460 USA; 28 Spelman College Department of Political Science, 350 Spelman Lane SW, Atlanta, GA 30314 USA; 29 Department of Geography, University of Georgia, Atlanta, GA USA; 30 Department of Political and Social Sciences, Graduate School of East Asian Studies, Freie Universität Berlin, Berlin, Germany; 31 Vulnerability and Human Condition Initiative, Emory University, Atlanta, GA 30322 USA

**Keywords:** Adaptation, Climate change, Horizon scanning, Mitigation, Research priorities

## Abstract

Climate change has far-reaching effects on human and ecological systems, requiring collaboration across sectors and disciplines to determine effective responses. To inform regional responses to climate change, decision-makers need credible and relevant information representing a wide swath of knowledge and perspectives. The southeastern U. S. State of Georgia is a valuable focal area for study because it contains multiple ecological zones that vary greatly in land use and economic activities, and it is vulnerable to diverse climate change impacts. We identified 40 important research questions that, if answered, could lay the groundwork for effective, science-based climate action in Georgia. Top research priorities were identified through a broad solicitation of candidate research questions (180 were received). A group of experts across sectors and disciplines gathered for a workshop to categorize, prioritize, and filter the candidate questions, identify missing topics, and rewrite questions. Participants then collectively chose the 40 most important questions. This cross-sectoral effort ensured the inclusion of a diversity of topics and questions (e.g., coastal hazards, agricultural production, ecosystem functioning, urban infrastructure, and human health) likely to be important to Georgia policy-makers, practitioners, and scientists. Several cross-cutting themes emerged, including the need for long-term data collection and consideration of at-risk Georgia citizens and communities. Workshop participants defined effective responses as those that take economic cost, environmental impacts, and social justice into consideration. Our research highlights the importance of collaborators across disciplines and sectors, and discussing challenges and opportunities that will require transdisciplinary solutions.

## Introduction

Climate change alters atmospheric chemistry and temperature, ocean chemistry and temperature, the frequency and intensity of extreme weather events, and precipitation patterns, all of which will directly and indirectly influence ecological systems and human well-being globally (IPCC 2014; Melillo et al. 2014). Patterns of climate change may differ at regional and local geographic scales (Ruth and Ibarraran 2009) and variability in susceptibility to harm, in ecological, social and infrastructure systems, will lead to differential impacts from climate change (Crimmins et al. 2016; Patz et al. 2005; Thornton et al 2014). Within the USA, for example, economic damages from climate change under a business-as-usual emissions scenario are projected to be strongest in southeastern states (Hsiang et al. 2017), where higher temperatures (Shepherd and Knox 2016), sea level rise (Neumann et al. 2015), and reduced water availability (Sun 2013) are forecast (Carter et al. 2014).

Society’s response to climate change is shaped by economic, social, ecological, and political factors (Melillo et al. 2014; National Academies of Sciences, Engineering, and Medicine 2016). At the local level, the impacts of climate change are most likely to be relatively high in regions and communities with relatively low levels of economic wealth, educational attainment, or social cohesion (Rufat et al 2015; Cutter et al 2003). In the southeastern USA, the State of Georgia is a valuable focal area for study because it contains multiple ecological zones (from the coast to the Appalachian Mountains) and varies greatly in land use, economic activities (e.g., agriculture, forestry, and urban-based industry), and social and political perspectives. The State already has a variety of potential responses to climate change in place, and communities across Georgia are actively exploring strategies to respond to climate change (Evans et al. 2016; Gambill et al. 2017). Some are working to assess vulnerabilities and build resilience to potential impacts (e.g., Evans et al. 2014), while others are developing technologies and policies to reduce emissions (City of Atlanta 2015). Several large Georgia-based multinational corporations are also proactively developing strategies to minimize the extent and effects of climate change and to create new economic opportunities in response to climate change (e.g., Anderson and White 2009).

Effectively responding to climate change will require cross-sectoral and cross-disciplinary perspectives both to develop effective technical and policy responses and to understand how such responses may lead to synergistic or antagonistic outcomes across regions and sectors (Mauser et al. 2013). To inform regional responses to climate change, private, non-profit, and government decision-makers at all levels need credible and relevant information from across the natural, applied, and social sciences. We seek to fulfill that need by identifying the key research questions that, if answered, could lay the groundwork for Georgia to take effective, science-based climate action at the State and local levels. The output we report on here, the Georgia Climate Research Roadmap (GCRR), is an initiative of the Georgia Climate Project (www.GeorgiaClimateProject.org), a network of colleges and universities working with federal, state and local government officials, non-governmental organizations, private industry, and other partners seeking to improve understanding of climate impacts and solutions in Georgia.

A bottom-up participatory approach was adopted to identify those key research questions necessary to take effective, science-based climate action in Georgia (Sutherland et al. 2011). This methodology has been used extensively in recent years to identify important research questions in a variety of fields, typically at a national or international scale or on a sectoral basis (e.g., Sutherland et al. 2009; Pretty et al. 2010; Fleishman et al. 2011; Boxall et al. 2012; Green et al. 2017). To our knowledge, this is the first time this methodology has been applied across sectors to address state-level research priorities. This approach involves, first, a broad solicitation of candidate research questions and, second, a systematic winnowing of those candidate questions by a group of experts, usually at an in-person workshop (Sutherland et al. 2011). Besides identifying important research questions, such exercises can serve to help improve clarity surrounding relevant policy questions, ensure that scientific research is aligned with policy- and decision-makers’ needs, and build new collaborative networks for research and information exchange (Rudd 2011). Specifically, the GCRR, through a multi-stakeholder process, sought to identify climate research questions whose answers would most benefit policy-makers, practitioners, and scientists in Georgia.

## Methods

For the GCRR, we followed the methodology outlined by Sutherland et al. (2011). First, we formed a 12-member steering committee, consisting of representatives from Georgia universities (Emory University; Georgia Institute of Technology; Georgia State University; University of Georgia), State and municipal government agencies (Atlanta Regional Commission; Georgia Department of Natural Resources; Georgia Sea Grant), the private sector (Coca-Cola; Smith, Gambrell, & Russell; WSP USA), and non-profit organizations (Second Nature; Southface Energy Institute). During the period from March 13th to May 5th, 2017, we solicited candidate research questions widely from individuals and organizations via a dedicated question submission website.

A workshop at Emory University (May 22–23, 2017) brought a group of experts together to discuss and vet the submitted candidate research questions. Attendees included steering committee members, invited workshop participants not previously involved in project design or implementation, and student assistants. In an effort to ensure that a wide diversity of research priorities were addressed, we strategically invited workshop participants to balance, to the best of our ability, sectoral, regional, disciplinary, and demographic characteristics across a group of approximately 40 participants. The workshop participants (co-authors on this paper) represented a broad distribution of interests, expertise, sectors, career stages, and geographic locations (supplementary information Table S1), helping to ensure that a diverse range of candidate questions and that other important questions not represented in the candidate question pool were considered.

The workshop followed the standard format (Sutherland et al. 2011), with an opening plenary that explained the context and workshop procedures, a series of nine breakout sessions (three parallel sessions of three topically-oriented breakouts each), and a closing plenary where the list of Georgia’s 40 important research questions was finalized. During the breakout sessions, participants considered groups of 12–16 candidate questions and identified two primary and two back-up questions for further discussion in the final plenary session. Breakout group participants were free to combine submissions, to draw core ideas from candidate questions and reword them, or to propose entirely new questions. At the plenary, a discussion among all workshop participants narrowed the list of candidate questions to a final list of 40 questions.

## Results

A total of 180 valid candidate research questions were collected from 132 submissions (see supplementary information Table S2). The final selection of key climate research questions important for policy- and decision-makers, practitioners, and scientists in Georgia is presented below grouped by theme. Each theme contains questions regarding both the effects of climate change in Georgia and potential responses. It is important to note that many of these questions cut across topical areas. Georgia’s 40 important research questions are not presented in rank order; a follow-up survey project (e.g., Rudd and Fleishman 2014; Rudd et al. 2014) will be used to rank the 40 questions and solicit further input on important research topics that may not have been covered at the workshop.

### Weather and climate

1. *What short-, mid-, and long-term climate impact scenarios should Georgia be planning for?*
  The impacts of future climate change on Georgia are likely to be diverse. Climate models project increases in the future frequency and/or magnitude of hazards (Melillo et al. 2014; Walsh et al. 2014) and downscaling from global models can provide crucial information at regional levels (Hay et al. 2002; Trail et al. 2013). To support effective planning and efficient investments, research is needed to quantify the most likely levels of impact and their upper and lower extremes for particular regions within the State. At the same time, planning horizons must consider that climate change impacts will accumulate through time, requiring the optimization of near-term investments to mitigate near-term risks (e.g., for a 10-yr time horizon) while recognizing the need for adaptability of longer-term investments to mitigate longer-term (e.g., for a 50-yr time horizon) climate change-related risks.
2. *How can signals of climate change be identified given Georgia’s exposure to a full spectrum of weather hazards?*
  In concert with accelerated warming, Georgia and the Southeastern USA are projected to experience more intense heat waves and droughts, more frequent and extreme flooding (Moore et al. 2014;), and stronger hurricanes (Ingram et al. 2013). Georgia, like much of the Southeast USA, experiences the full range of extreme weather events (Emrich and Cutter 2011), so regional vulnerabilities to specific types of events need to be identified. Understanding how particular types of climate change events manifest is necessary to develop adaptation and mitigation strategies and investments to deal with risks that society and institutions face due to changing exposure to weather hazards (National Academies of Sciences, Engineering, and Medicine 2016). In particular, Georgia’s coastline represents an area of compound risk from climate impacts, given the high level of certainty that sea levels will rise and the dramatically increased levels of risks associated with storm surges in coming decades (Carter et al. 2014).
3. *What are the appropriate physical parameters and corresponding observational data sets needed to properly characterize Georgia’s risk and vulnerability to weather hazards likely associated with climate change?*
  Risk is often described as some function of exposure to weather-climate hazards (e.g., droughts, floods, storms, and temperature extremes), the vulnerability or sensitivity of people, infrastructure and ecosystems exposed to those hazards, and their resilience (KC et al. 2015; Kreft et al. 2014). To properly quantify the risk that Georgia faces at meaningful spatio-temporal scales, biophysical and social data, including robust and long-term meteorological, climatological, demographic, and economic datasets and projections, will be required. In some cases, new observational or modeling capabilities (Maurer et al. 2014) will also be needed to fill data gaps.
4. *What are best practices in planning for and responding to extreme weather events and hazards, and how can Georgia improve these practices going forward?*
  An analysis of current practices and gaps in the field of climate adaptation is needed to build an understanding of what is being done for and by communities across the State, building on and updating previous guidelines (e.g., Georgia Department of Community Affairs et al. 2014) to develop a current set of best practices. Such an analysis must include urban and rural, coastal and inland settings, and include consideration of impacts and responses targeted at the state’s most vulnerable populations, guided by lessons from recent weather-related disasters in the southeastern US. For example, population displacement of vulnerable citizens and long-term impacts on state economies can be quantified for specific hurricanes (e.g., Harvey, Irma, and Maria), and mitigation plans constructed accordingly.
5. *How has the changing risk of extreme weather events and shifting baselines impacted short-, medium-, and long-term costs of climate change, and what are the implications of these changes?*
  Information on and perceptions of “normal” climate conditions shape decisions and actions on a wide range of topics across the state, including energy and water infrastructure, agriculture, public health, natural resources, and local planning. Georgia is projected to transition towards more extreme climatic conditions on a number of fronts (Melillo et al. 2014), including the frequency and intensity of heavy rainfall events (“low to medium confidence”), drought conditions (“low to medium confidence”), extreme heat events (“high confidence”), and coastal flooding (“high confidence”). As these baselines shift, it may be useful to pursue corresponding shifts in city to state-level infrastructure planning (e.g., urban drainage infrastructure; Kang et al. 2016) and regulatory frameworks (e.g., mandatory planning standards based on the level of a “500-yr-flood”). To facilitate this assessment, a systematic examination within and/or across sectors and geographies may yield insights into the environmental data underpinning current decision-making and existing policy, and recommend updates to those planning points to conform with projected environmental conditions.
6. *How do urban landscapes directly affect local-to-regional weather and climate processes?*
  Anthropogenic climate change is often narrowly framed in terms of greenhouse gas (GHG) emissions warming the planet. However, the National Academies of Sciences, Engineering, and Medicine (2016) recently laid out the various ways that urban landscapes further change weather and climate processes. While urban heat islands and pollution are the most familiar, other pathways also exist, such as the development of storms, flooding, and changes to carbon and nitrogen cycles (Seto and Shepherd 2009; McLeod et al. 2017). As Georgia becomes increasingly urbanized, urban-weather-climate interactions will increase levels of stress on ecosystems, infrastructure, and society, implying the need for new thinking in urban planning, policy, management, and design.

### Ecosystems in Georgia

7. *What species and ecosystems are most at risk of population declines or extirpation due to climate stresses?*
  Species distribution models have been used to predict climate-driven habitat shifts for a variety of taxa (Jeschke and Strayer 2008; Kearney and Porter 2009), including mechanistic models that rely on physiological data to predict species survival and evolutionary fitness in a range of environmental conditions (Chuine et al. 2000). These models, however, require a complex understanding of physiological characteristics of the species in question, few of which have been published to date. In the Georgia context, the 2015 State Wildlife Action Plan (Georgia Department of Natural Resources 2015) identifies several priority conservation actions for climate change adaptation for avian, terrestrial, and aquatic species, and the Georgia Department of Natural Resources is supporting further research into coastal species vulnerability. A further evaluation of existing data, tools, and research would add value, and further research is needed to improve predictive models of species decline and risk over time, and determine likely outcomes for ecosystems.
8. *How will the effects of multiple human-induced stressors affect species distribution and biodiversity change across Georgia?*
  Changes in species composition caused by climate change may affect Georgia’s terrestrial and aquatic ecosystems, influencing biodiversity, species interactions, and ecosystem processes. Species distributions across latitude and altitude are changing globally as a result of climate change (e.g., Parmesan 2006; Møller et al. 2008; Burrows et al. 2011). The species gained and lost may bring new challenges, such as the introduction of new pathogen and pest species (Cable et al. 2017). Further, these changes are occurring in the context of other human-caused stresses, such as habitat loss and pollution, which may exacerbate climate-related stresses (Holyoak and Heath 2016; Noyes et al. 2009). A summary of existing data, tools, and research on this topic in Georgia can further inform decisionmakers and can serve as a basis for further research.
9. *What are the most ecologically sound and cost-effective options for terrestrial ecosystem management to reduce the effects of climate change on ecosystems?*
  Many strategies are possible for managing terrestrial farmlands, wetlands, and forests, emphasizing different ecological traits and management tools. For example, some strategies emphasize protecting habitats or ecosystems of high importance, while others emphasize the preservation of connectivity, which would allow species to migrate through landscapes of mixed land use as the climate changes. The management of Georgia ecosystems can potentially be financed using a variety of methods (e.g., tax incentives, payments for ecosystem services, land purchases, encouraging or regulating agriculture and forestry best management practices), but such methods may vary greatly in cost and levels of acceptability for various management agencies, stakeholders, and citizens. Information about the trade-offs that groups are willing to make in terms of costs and management outcomes is needed to help evaluate the appropriateness of different management options (e.g., Nalle et al. 2004).
10. *What ecosystem services are most at risk in Georgia due to climate change?*
  Ecosystem services are the ecological goods and services that benefit humans (Millennium Ecosystem Assessment 2005): some type of ecological product or service (e.g., water purification, carbon storage, food, timber, recreational opportunity, presence of endangered species) must provide direct value to humans. Tenuous links often exist between environmental stressors caused by climate change, changes in ecological structure and function, the production of valued ecosystem services, and the economic value of those services to diverse citizens and stakeholders (Balvanera et al. 2006; Weitzman 2012; Hejnowicz and Rudd 2017). In Georgia, further research is required to categorize ecosystem services that provide benefits to humans while assessing the risks they face due to climate change.

### Oceans and coasts

11. *How will changes in abiotic conditions in the ocean influence Georgia’s climate and coastal ecosystems?*
  Abiotic conditions, including physical and chemical properties of the oceans, are changing (Caldeira and Wickett 2003; Doney et al. 2012) and are already significantly affecting biological and human systems (Poloczanska et al. 2013). Increasing sea surface temperatures can exceed species tolerances and induce migration of species to cooler waters or cause extirpations of populations at the geographic fringes of a species range (Poloczanska et al. 2013). Temperature increases can also influence stratification, limiting access to light or nutrients and, consequently, coastal productivity and low oxygen conditions in coastal waters (Meire et al. 2013). Changes to temperature (Balmaseda et al. 2013) and salinity may also alter ocean circulation patterns in the future, affecting marine and human systems (Broecker 1997). In Georgia’s coastal waters, understanding the potential effects of changing abiotic conditions provides information necessary for developing strategies for maintaining coastal ecosystems and economic activities in the face of change.
12. *How will climate change affect coastal biodiversity, ecosystems, economy, and ecosystem services?*
  Changes to coastal productivity, species diversity, and ecosystem function will affect human communities that depend economically on coastal resources (Barbier et al. 2011). Georgia’s Atlantic coast has barrier islands, estuaries, and some of the most expansive marshes in the USA. These areas are facing sea level rise and an increase in coastal flooding from extreme weather events such as Hurricanes Matthew and Irma. While there are projections available for sea level rise (Parris et al. 2012), it is not specifically known how various aspects of environmental change will alter Georgia’s coastal ecosystems, their biodiversity, and the ecosystem services they deliver. Identifying climate-related impacts along the Georgia coast is especially important as we evaluate adaptation options designed to protect coastal communities. For example, coastal armoring can affect species assemblages, connectivity, and other functions of intertidal habitats (Dugan et al. 2017).
13. *What are the most ecologically sound, cost-effective, and just adaptation options to address coastal hazards due to climate change?*
  Georgia faces increasing exposure to intensifying and compounding coastal hazards, such as extreme weather events, flooding, erosion, storm surge, and saltwater intrusion (Williams 2013). Both State and local communities need to be engaged, as some solutions may require a combination of long-term infrastructure investments, as well as local adaptations to changing conditions and risks at the land-sea interface. Local communities need data and research on the best methods to adapt to these challenges, while balancing economic development, environmental integrity, population growth, equity for citizens, and managing technical and human capacity constraints (e.g., Arkema et al. 2013). Because local governments often do not have the capacity to use raw research and scientific tools, one-on-one technical assistance will help local governments to integrate long-term adaptation methods into required planning processes in Georgia (e.g., Capital Improvement Plans, Hazards Mitigation Planning, Disaster Recovery and Redevelopment Planning).
14. *How will sea level rise and flooding affect the economy of coastal Georgia?*
  Sea level rise and the associated increased frequency of coastal flooding due to decreased efficiency of stormwater management systems during high tides is already impacting coastal communities in Georgia (Evans et al. 2016). To adequately plan for adaptation strategies and investment, reliable data are needed on the increased risk associated with flooding and storm events at local levels, including economic effects on sectors such as tourism and recreation, fisheries and aquaculture, real estate values, and the price of property insurance. Communities will also need to know how flooding will influence critical infrastructure such as ports, roads, health care facilities, and government buildings, as well as construction of new residential and commercial centers.
15. *How will climate-related changes in upstream water systems influence coastal ecosystems?*
  Dynamic coastal ecosystems are strongly influenced by the adequate quantity and quality of upstream freshwater surface flow and groundwater. Projections indicate that many of the world’s watersheds will experience dramatic changes in river discharge resulting from changes in drought, precipitation, and temperatures, and many will experience water stress (Palmer et al. 2008; Syvitski et al. 2005). These changes in riverine flow can impact the coast’s productivity in response to a change in the timing and amount of freshwater, nutrients, and sediment delivery (Alber 2002; Syvitski et al. 2005; Camargo and Alonso 2006). Higher water temperatures and changes in freshwater delivery will alter estuarine stratification, residence time, and eutrophication (Scavia et al. 2002). The effects of climate change on water systems and community water needs are unknown but expected to be problematic (Palmer et al. 2008) and also exacerbated by other ecosystem stresses (Scavia et al. 2002). The land adjacent to the coasts of Georgia is primarily agricultural, so coastal conditions will be influenced by agricultural practices and terrestrial adaptations to climate change in the agriculture sector.

### Agriculture, forestry, and food

16. *What are the economic costs and benefits of climate change for agricultural and natural resources?*
  Climate change is expected to affect agriculture and natural resources across the State in a variety of ways, including increases in the length of the growing season, decreased need for winter heating, increases in heat stress to crops and livestock, and changes in rainfall, humidity and evapotranspiration, which could lead to both more severe droughts and more floods (Melillo et al. 2014). The costs of these changes and how Georgians might reduce risk from environmental hazards while taking advantage of the potential benefits of a warmer climate are not well understood. Agriculture, the single biggest industry in Georgia and which in 2015 contributed $74.9 billion in output (8% of Georgia’s $917.6 billion economy) (University of Georgia 2017), is particularly at risk. For instance, a 2007 drought caused an estimated $787 million in agricultural production losses (Flanders et al. 2007). In 2017, blueberries and peach crops were impacted by unusually early spring warming followed by atypical mid-March frost event. The pecan harvest was negatively affected by the unusually strong inland winds of Hurricane Irma.
17. *How will climate change impact food security in Georgia?*
  Climate change could affect multiple links along food supply chains, including the viability of existing crops (Berardy and Chester 2017), the costs and accessibility of transportation channels (Chapman 2007), and patterns in consumer demand (Wicker and Becken 2013), as well as the prices for competitive products from other regions (Ziska et al. 2016). Besides food price and availability, climate-driven changes in food supply chains can affect public health due to their impact on food affordability. Information is needed to assess the current state of food security in Georgia, develop projections of future food supply and pricing scenarios, identify points of impact for vulnerable communities and citizens, and devise solutions that are locally significant, but can also be linked synergistically to other efforts in the State.
18. *How can the environment help Georgia adapt to climate change?*
  Ecosystem processes are critical to sustaining our communities and adapting to a changing climate (Kabisch et al. 2016). For example, green infrastructure has been shown to be a cost-effective approach for increasing climate resilience in both urban and undeveloped areas (Opperman 2014). Atlanta has adopted some infrastructure innovations (www.atlantawatershed.org/greeninfrastructure) but research is needed to identify how other cities and municipalities can use nature to not just efficiently increase resilience to various stressors. Coastal protection is another example where the effectiveness and value of coastal protection has been assessed for other regions (Narayan et al. 2016; Ruckelshaus et al. 2016). Georgia’s coast encompasses a series of barrier island complexes and salt marshes that protect the coastline from storm surges and wave action. These coastal ecosystems are threatened by rapid growth of coastal communities (Marine Extension and Georgia Sea Grant 2017). Improved understanding of the coastal resources and the ecosystems services in Georgia and the Southeast is needed to identify cost-effective green infrastructure approaches to adapt to a changing climate.
19. *What policies and practices could Georgia use to increase carbon sequestration in agriculture and forestry?*
  Increases in atmospheric carbon dioxide can be reduced by sequestering carbon in the environment through increased growth of trees, improvements in soil health, and reduction of losses from urbanization and draining wetlands (Lackner 2003; Lal 2004; Mitsch et al. 2013; Nowak and Crane 2002). Best management practices of farms, forests and other ecosystems can reduce the emission of carbon into the atmosphere, improve the uptake of carbon from the air by plants, and retain carbon over the long term (Snyder et al. 2009; West and Marland 2002; Yang et al 2008). Such practices have potential synergies (Paustian et al. 1998), helping to maintain soil quality, bolstering the financial viability of farms and agribusinesses, and maintaining ecological processes and ecosystem services, including protection of valuable and endangered species (Hampe and Petit 2005). Georgia’s forests and agricultural land already provide a net carbon sink of more than 50 million tonnes, and the potential development of future carbon trading markets (Sorrell and Sijm 2003) offer an incentive to understand how sequestering carbon efficiently can provide economic opportunities for Georgia businesses.
20. *What sources and forms of information and communication can be used to build resilience among farmers and farming communities faced with changing weather patterns and extreme events?*
  Agricultural communities in Georgia are very diverse, as are their products and customers (Crane et al. 2010; Furman et al. 2014). As weather becomes more extreme, tools and strategies to find, communicate, and use information are needed to build resilient systems. Several tools and communication technologies have been developed for this purpose (Garcia y Garcia et al. 2006; Paz et al. 2012). For example, the U.S. Department of Agriculture’s Climate Hubs (https://www.climatehubs.oce.usda.gov/) include research and tools on irrigation, animal agriculture, and forestry. There is considerable scope however for improving these technologies, adapting them for Georgia conditions, developing new tools, and/or or introducing them to growers not familiar with them. This implies the need for more explicit and participatory engagement strategies along food supply chains and methods that directly incorporate input from farmers and extension agents, to take into consideration Georgia farmers’ explicit interests, needs and ideas (Bartels et al. 2013).

### Water

21. *How do climate change projections affect scenarios of quantity and quality of future water supply for Georgia through 2050 at local and basin scales?*
  In 2017, Georgia revised 11 Regional Water Plans to help manage water resources in a sustainable manner through 2050. The plans (Georgia Environmental Protection Division 2017) identified gaps between projected water use across multiple sectors and modeled future surface water and groundwater capacities. Only one of those plans (Metropolitan North Georgia Water Planning District 2015) explicitly addressed climate resilience in its long-range planning. Other regions of Georgia would benefit from conducting appropriately scaled studies to assess how changes in patterns of precipitation will affect streamflows, groundwater availability, water quality, and water supply through 2050 and beyond. Advances in streamflow modeling (specifically correcting the known deficiencies in the unimpaired flow dataset) could help enhance water supply planning in the face of climate variability and change.
22. *What are the most effective data capture and reporting mechanisms for assembling a statewide comprehensive dataset to inform water planning in the context of climate change?*
  Comprehensive data and uniform reporting mechanisms are needed to develop an understanding of water supply and use, create early warning systems, and develop better management strategies in the context of climate change. There is, however, no statewide system that allows for local water resource managers to provide regular reporting. To maintain this kind of data repository, long-term commitments for regular data entry would need to occur at the local level. These data and their reporting mechanisms should derive from various sectors including municipal, industrial, energy, agriculture and other sources. For greatest benefit, the system would need capabilities to aid the end-user and the responsible manager. The creation of this system would provide near-term and long-term benefits for exploring the effects of climate change and climate variability on Georgia’s water resources.
23. *What are the most effective and efficient investments to improve resilience of potable water infrastructure under different and uncertain future climate and water use scenarios in Georgia?*
  Major water infrastructure projects are typically very expensive to build. While no government would want to have insufficient infrastructure to provide its citizens with clean water, it is also possible to overinvest in infrastructure when alternative lower cost interventions (Gleick 2003) are possible. However, uncertainty regarding precipitation patterns, as well as urban development patterns, consumers’ willingness to engage in water conservation measures, and water price will influence infrastructure investment choices. Collaborative work between water managers and researchers can identify high risk future scenarios requiring large infrastructure investment as well as robust low-cost measures that would preclude the need for overinvestment in infrastructure (Groves et al. 2008; Lempert and Groves 2010). Some water utilities and planning districts in Georgia have conducted research into the potential impacts of climate change; for example, the Metropolitan North Georgia Water Planning District conducted a Utility Climate Resilience Study (Metropolitan North Georgia Water Planning District 2015) and the Brunswick-Glynn County Joint Water and Sewer Commission conducted a climate resilience assessment in 2017. Other water utilities could benefit from similar research to identify key risks to water supply, assess the robustness to uncertainty of different investments and interventions, and make explicit the assumptions regarding demographics, climate change, and consumers’ water use behavior.

### Energy and transportation

24. *What are the optimal strategies to reduce greenhouse gas emissions through energy efficiency and renewable energy at state and regional levels in Georgia, and what are appropriate targets that correspond to these strategies?*
  The energy sector is a key contributor to GHG emissions and accordingly is a considerable source for potential emissions reductions. Several states and municipalities across the United States have established targets for deployment of renewable energy and energy efficiency (e.g., NC Clean Energy Technology Center 2017). Any future consideration of such targets in Georgia can be informed by a thorough technical and economic analysis to better understand least-cost strategies. This work could be undertaken either as part of a comprehensive assessment of statewide GHG reduction options (such as that outlined in question 39) or as a stand-alone exercise, such as that undertaken by North Carolina in 2006 (North Carolina Utilities Commission 2006). Both efficiency and renewable energy policies may have co-benefits that may not be fully and clearly accounted for in the absence of a comprehensive assessment.
25. *What are the costs and benefits of changes in Georgia’s transportation systems and how will those changes impact greenhouse gas emissions?*
  The U.S. transportation system is experiencing a period of dramatic technological change. New sources of propulsion, vehicle control, and system management have been developed across the nation (Anderson et al. 2016), and the transportation industry and profession is predicting substantive changes to transportation systems over the coming 30 years due to autonomous and connected vehicle operations (Fagnant and Kockelman 2015). Such changes could, in the short term, affect the manner in which people and freight movers use the transportation system, thus affecting emissions and energy use. Over the longer-term, these technologies and system management strategies could affect where and how people live, thus influencing other aspects of the environment. A long-term vision of how the transportation system for Georgia will operate and be used is needed to assess the likely consequences to GHG emissions (Bigazzi and Figliozzi 2013). Research is needed to examine the broader societal questions arising from these changes, for example the equity impacts on different Georgia populations, the impacts on rural areas versus urban areas, and additional economic costs associated with new forms of movement for people and goods.

### Human health

26. *What are the most significant climate-related health threats for communities in Georgia?*
  Climate change will lead to alterations in the patterns (intensity, frequency, spatial distribution) of many climate-sensitive environmental health exposures (temperature, precipitation, extreme events, etc.). These exposures, individually or in combination, will impact a range of health outcomes through direct and indirect pathways (McMichael and Woodruff 2005; Portier et al. 2010; Crimmins et al. 2016). While excess deaths and illness during a heat wave is an example of a direct health impact (Stone et al. 2014; Beard et al. 2016), changing patterns in seasonal temperature and precipitation can alter habitats for certain ticks (e.g., *Ixodes scapularis*, linked to Lyme disease) thus indirectly spreading vector-borne diseases (Ostfeld and Brunner 2015). Different methodologies exist to link climate and health data to assess the range of epidemiological risk associated with climate change (World Health Organization 2014) but specific research is needed to assess threats in Georgia.
27. *What health risk management strategies are most effective for addressing shifting climate-related health vulnerabilities among various communities in Georgia?*
  There are many potential health effects of climate change in Georgia, ranging from increased heat-related illness to shifts in vector-borne disease (Schramm 2013). Research is needed to examine the scope and scale of these impacts across different parts of the state, to document current methods being employed to prevent negative health outcomes, and to determine the most effective strategies for managing the health risks of climate change in the future.
28. *How can the economic implications of the health impacts of climate change in Georgia best be quantified and communicated to decision makers?*
  The economic implications of climate-related health impacts may be considered from the perspective of health damage costs (Haines et al. 2006), impacts on healthcare infrastructure (Bell et al. 2016), or costs and benefits of adaptation strategies designed to mitigate the adverse health impacts (Martinez et al. 2015). Such economic estimates are critical for decision-makers trying to prioritize resource allocation decisions across a range of climate adaptation and mitigation choices. The inclusion of economic estimates in framing climate risks for effective decision-making is part of ongoing research (Stern 2007).
29. *What are the most effective methods in Georgia to build resilience in the healthcare and public health systems to protect at-risk communities from climate change?*
  Public health programs (implemented by county and state health departments) and the healthcare sector (hospitals, nursing homes, etc.) will be critical in responding to the health impacts of climate change. Enhancing the existing capacity to respond to climate-related health events and building the resilience of communities to avoid negative health impacts could play a major role in protecting at-risk citizens and communities. Adaptation and interventions exist to protect at-risk communities (Anderson et al. 2017), but it is unclear which of those would be most useful for communities across Georgia.

### Communities and infrastructure

30. *How will climate change in Georgia create risks that impact urban and rural infrastructure and the resilience of human populations?*
  Extreme weather and longer-term changes in climate can significantly affect the resilience of infrastructure to external influences (Neumann et al. 2015; Bell et al. 2016). Given the reliance of communities on functional energy, water, and transportation infrastructure, disruptions to infrastructure systems could have a negative impact on human population resilience. The consequences of potential disruptions would vary in urban and rural areas, and by the connectivity and interdependence of the infrastructure systems (Olsen 2015). For example, increasing droughts could have significant effects on human health and community well-being. Roads or railroads disrupted due to storm-related impacts could affect personal mobility, economic supply chains, and the ability to access affected areas. Research would help build understanding about the linkages between expected climate change-related risks and the critical infrastructure and population support systems that form the basis of the quality of life for Georgia’s citizens.
31. *How will local, regional, and international displacement of people by climate change-related events affect Georgia?*
  Weather events and their hydrological impacts, including those projected to become more frequent and/or intense with a changing climate, can drive significant displacement of people (IPCC 2012), as can longer-term trends like sea-level rise (Hauer et al. 2016). As the existing human habitat patterns change, nuances of population needs, vulnerabilities, demographics, and concentrations will impact state resources and infrastructure in ways not yet fully known or understood (Hauer 2017). On the forefront of these impacts will be local governments and service systems which, in turn, will look to regional, state, and federal governments for guidance and support. Some Georgia communities may see an increase in population, and others a decrease; those impacts will have different implications for regional and state resources. Understanding of the resource and infrastructure stressors is therefore critical for advance preparation at all levels of Georgia government and service provision.
32. *How can cities and counties decrease greenhouse gas emissions and adapt to the coming climate from an urban planning and design perspective?*
  Land use patterns and built environments, and the infrastructure that supports them (such as transportation), directly influence the levels and concentrations of GHG emissions (Transportation Research Board Institute of Medicine 2005). Using tools such as comprehensive planning, zoning, tax incentives, and negotiated agreements with developers, communities can influence where development occurs and in what form (Meyer and Dumbaugh 2004). Development patterns can also strongly influence how transportation systems and other infrastructure systems are used, further impacting future GHG emissions. Therefore, it is necessary to undertake more research to understand how communities should adjust their existing and planned infrastructure to account for these impacts. There is a need to identify which GHG-reducing and adaptation methods and tools for implementing these strategies should be available to Georgia communities, and the likely benefits and costs associated with each.

### Human values, social equity, and environmental justice

33. *How do beliefs, attitudes, perceptions, and knowledge about climate change and its potential solutions vary across Georgia?*
  People’s perceptions, attitudes, and behaviors related to climate change are often influenced by a variety of factors that result in differences of opinions regionally and by demographic groups (Etkin and Ho 2007; Lujala et al. 2015). Early research demonstrated that there were notable regional variations in beliefs within Georgia (Howe et al. 2015), but a more in-depth statewide analysis could provide valuable information about how different stakeholder groups perceive climate change and value responses / approaches for addressing it. This information could assist decision makers, scientists, and educators as to the potential effectiveness of public awareness and education campaigns, and to help identify stakeholders most supportive of mitigation solutions and/or adaptation strategies.
34. *What are the appropriate datasets and methods to assess the susceptibility of at-risk communities in Georgia to the impacts of extreme weather and climate change?*
  There is an extensive body of scientific studies that document that people of color and low-income people live in communities that are adversely impacted by a disproportionate level of environmental pollution (Bullard 2008; Collins et al. 2016; Mohai and Saha 2007; Mohai et al. 2009). Climate change could lead to increasing exposure to hazards among these communities, exacerbate existing vulnerabilities, and hinder their capacity to cope (United Nations Human Development Programme 2007; United Nations Human Development Programme 2014; Gamble et al. 2016). Some data relevant to assessing susceptibility, vulnerability, and resilience are available through traditional sources and are reported by statistical agencies, but other data are also necessary. Generating the most relevant data may require developing collaborative data collection platforms with stakeholders in local communities.
35. *What are the most effective and practical ways to frame and communicate the challenges and opportunities of climate change in Georgia?*
  In 2016, 68% of adult Georgians thought global warming was occurring, and 51% believed that it was caused by human activity (Marlon et al. 2016). Framing (Nisbet 2009) and communicating the issues associated with climate change is critically important to the future of climate-related action and policy in Georgia. Finding ways to frame and communicate the issues associated with climate change could have impacts on the way the State and its citizens react to the effects of climate change. Communication of the causes and consequences of climate change in Georgia is needed for the public and for policy- and decision-makers.
36. *What are challenges and opportunities in creating evidence-based K-12 and post-secondary climate change curricula for different types of institutions and regions?*
  Leiserowitz et al. (2017) recently found that by a three-to-one margin, Americans want schools to improve climate change education. Educators in K-12 schools face a challenge in delivering evidence-based information about climate change at an appropriate level of sophistication due to the complexities of Earth system science, political and corporate influences, and social norms arising from strongly held opinions and beliefs of parents and/or administrators. Mitchem et al. (unpublished data) found that the type of K-12 institution attended (public, private nonreligious, private religious, or home school) influences Georgia students’ opinions about climate change, consistent with other work on student beliefs (Trautwein and Lüdtke 2007). There are opportunities to narrow the gap between scientists and the public by increasing education on this topic using educational standards, scientific sources, and lesson plans created with climate scientists’ input (Perkins et al. 2016; Ranney and Clark 2016).
37. *How do equity and justice implications of reducing greenhouse gas emissions vary for alternative policy and technology strategies?*
  Addressing climate change through mitigation or adaptation strategies often poses tradeoffs relating to the efficiency and equity of policy outcomes (e.g., Böhringer et al. 2012). For example, mitigation policies that raise the price of electricity and heating fuel by promoting renewable energy may disproportionately increase energy cost burdens of low-income households (Johnson et al. 2017). Similarly, programs designed to address these burdens may only be accessible to certain populations in Georgia. Policies that attempt to influence transportation decisions by encouraging alternative fuel vehicles, increased public transportation supply and accessibility, or other options will have similar distributional consequences. In addition to assessing potential burdens among citizens and communities, it is important to assess potential co-benefits (e.g., health, innovation, education, job creation) of various policies and technological innovation strategies. The tradeoffs and consequences of policy options must be properly understood if technically, economically, and politically acceptable interventions are to be taken.

### Mitigation and adaptation across multiple sectors and scales

38. *What are immediate steps that policy-makers at all levels can take to implement climate mitigation and adaptation solutions?*
  For policymakers across Georgia to take effective, science-based climate action, it will be necessary to synthesize and integrate findings from important research questions into succinct synopses of action steps at the state, county, municipal, community, and/or organization level. Given the historical preference of policymakers to identify a short list of priorities for their first weeks or months in office, it is likewise desirable to present “low-hanging fruit” and “quick win” opportunities for climate action with potentially positive and attractive outcomes. This implies a need for a comprehensive comparison of costs and benefits not only for various strategies for addressing particular issues, but also a high-level scan of the relative merits (potentially political as well as economic) across Georgia’s important climate research questions.
39. *What are the most cost-effective and equitable policy and technology options for reducing emissions of greenhouse gases in Georgia, and how can these be prioritized?*
  Several systematic evaluations of emission reduction opportunities have been conducted at a global scale (Nauclér and Enkvist 2009; Hawken 2017). The Paris Agreement under the United Nations Framework Convention on Climate Change called for the development at the national level of “long-term low GHG emission development strategies” to identify pathways for reducing GHG emissions while sustaining economic growth (United Nations 2015). The United States and several other countries have developed such strategies (White House 2016), but there is no similar strategy for Georgia. Other jurisdictions have made progress at the State level: the Regional Greenhouse Gas Initiative (www.rggi.org) in the northeast USA, for example, agreed in August 2017 to reduce GHG emissions by 30% across 9 States by 2030. Common approaches for assessing and presenting emissions reduction options include marginal abatement cost curves and stabilization wedges (e.g., Blok et al. 2012; Barker et al. 2016), as well as methodologies for incorporating co-benefits into these analyses (e.g., Ürge-Vorsatz et al. 2014). Assessments of options can then be used to prioritize actions and/or develop action plans. It will be important to factor equity considerations into such a prioritization and/or planning process.
40. *Which policies, regulations, and practices are most effective at different levels of governance for climate adaptation and mitigation?*
  Efforts to address climate change and its impacts in systems with many decision-makers who are subject to different (and sometimes conflicting) incentives, policies and regulations, and operating at multiple scales presents a challenge to policy analysts. In Georgia, it would be useful to assess which interventions or investments are most effective at which scales (e.g., international, national, state, regional, local, organizational, and/or individual) and identify policy pathways that are effective and adaptive, retaining flexibility to respond to changing conditions in the future. The simultaneous growth of adaptation and mitigation legal regimes may facilitate a reasoned and effective response to climate change. Legal solutions can help ensure that local, state, and federal governments, regional compacts, organizations, and individuals both limit climate change (through mitigation) and better respond to present and future climate risks (through adaptation) when making decisions about the built and natural environment. Existing laws could be modified or new laws crafted to deal with climate change (Gerrard and Kuh 2012) and ensure the equitable distribution of the benefits of both climate change adaptation and mitigation (Ruhl 2010).

## Discussion

Through this process, we identified 40 research questions important to supporting effective decision-making as climate change manifests in Georgia. Our participatory, cross-sectoral process was intended to ensure that the 40 questions we identified cover the spectrum of issues likely to be important to policy- and decision-makers, practitioners, and scientists in Georgia. We believe that our State-level exercise provides a useful example of the process of research question identification for other coalitions interested in identifying climate change research priorities at a sub-national level. While each area will have unique questions they must deal with, the process would be similar, involving engagement of experts from across disciplines and sectors in a way that provides novel networking and learning opportunities. Beyond the substantive issues that each of Georgia’s Top 40 climate questions raise, it is also possible to identify five cross-cutting themes relating to climate change in Georgia.

### Broad themes

#### Data requirements and methods for monitoring climate change and its effects

Five of the questions in the GCRR relate to specific data and/or methodological requirements for monitoring climate change and its effects in Georgia. Robust data will be essential to providing effective answers to many other GCRR questions as well. The State needs information on a variety of physical and ecological parameters in both terrestrial and marine environments to identify the different ways in which climate change manifests in Georgia and to distinguish between enduring changes in climate and more transient conditions caused by normal fluctuations in weather. Ecological monitoring is needed both to identify the ‘fingerprint’ of climate change and to help identify the species and habitats that are most at risk from changes in the biophysical environment. Data will further be needed to link climate change effects to social and economic consequences that are salient to decision-makers and citizens. Data collection has been identified as a key priority in a prior USA biodiversity-oriented research prioritization exercise (Fleishman et al. 2011). However, long term data collection can be challenging as funding for these projects can be hard to maintain, often because this type of research can be viewed as mundane. More significantly, data collection is a direct cost to government departments and municipalities, which can leave it vulnerable as an expendable target as agencies and departments face budget constraints.

#### What are the impacts of climate change in Georgia?

Twenty-one of the GCRR questions are about the current and potential physical, social, economic, and ecological impacts of climate change, including weather hazards, ecosystems and ecosystem services, coastal ecosystems and economies, agriculture, natural resources, food security, water supply, health, infrastructure, and at-risk communities. Though there have been multiple international and national assessments of climate impacts (e.g., Melillo et al. 2014; IPCC 2014), there have been relatively few systematic reviews of climate impacts at a state level in the United States, with one notable exception being NOAA’s 2017 Georgia State Summary (Frankson et al. 2017). New research into the GCRR questions about climate impacts, as well as further work to synthesize what is already known about these questions, could significantly benefit policy- and decision-makers as well as practitioners across the state

#### Developing effective responses to climate change

Eighteen of the GCRR questions relate to solutions to climate change challenges. Of these, nine focus on adaptation measures, five focus on mitigation measures, and four contain elements of mitigation and adaptation. Some of these questions focus in on particular sectors and themes, including agriculture, water, health, energy, transportation, and infrastructure. Others cut across multiple sectors and geographic scales. Responding to climate change at the State level will involve a variety of actions and strategies that include mitigation and adaptation efforts, and in some cases efforts that combine mitigation and adaptation. Innovations in technology or policy in one sector will influence other sectors, so challenges related to the synergistic or antagonistic effects of different responses will be important to identify solutions that can be both viable and self-sustaining over time. Georgia will need to plan for and cope with changes in social structures and processes, changes in global as well as national climate change policy, and long-term infrastructure investments.

#### The role of environmental justice when considering climate change impacts and solutions

Six of the GCRR questions and their descriptions explicitly mention issues relating to equity, environmental justice, and/or at-risk communities. Furthermore, multiple participants at the workshop emphasized the importance of integrating these considerations throughout climate research, policy, and practice in Georgia. Many characterized effective responses as those that take economic cost, environmental impacts, and existing vulnerabilities into consideration. Very often economically impoverished and politically disenfranchised communities lack resources required for adaption and building resilience to the shocks of climate-related and extreme weather events. Additionally, some communities may be poorly equipped or lack institutional access to respond to the effects of climate change due to both their increased exposure to hazards (e.g., coastal towns exposed to storms, towns in northern Georgia exposed to forest fires, and urban neighborhoods suffering from heat islands and poor housing) and susceptibility to damages. Efforts to addressing environmental injustices must consider who is most adversely impacted by environmental hazards and how their response is shaped by the various resources that different groups have access to (National Research Council 2011).

#### Considerations of geographic scope and scale

During the workshop, consideration of the GCRR questions frequently returned to issues related to geographic scope and scale. Though many questions are posed at the State level, all acknowledged the importance of focusing some analyses and solutions at the regional or community level and/or in specific geographies. Nine GCRR questions explicitly refer to a scope smaller than the State level, and five of the questions focus on the Georgia coast as one particular location of interest. Regarding the community scale, workshop participants discussed the value of looking at considerations unique and/or pertinent to both urban and rural communities. Finally, one GCRR question explicitly focuses on the issue of geographic scale, exploring what solutions are most effective at different levels of governance.

### Importance of transdisciplinary research and solution building

A changing climate influences many facets of society and presents pressing research questions for diverse fields of study. Addressing the challenges of climate change will require expertise from multiple academic disciplines and from across multiple sectors of society, engaging public, private, and non-profit organizations (Dilling and Lemos 2011; Weaver et al. 2014). This cross-disciplinary and cross-sectoral combination is usually referred to as transdisciplinary research (Lang et al. 2012). Our workshop highlighted the importance of having research participants from across disciplines and sectors, serving not only as a forum to identify the top priorities for Georgia, but also to discuss challenges that will by their very nature need transdisciplinary solutions.

### Validity

Two important considerations in all bottom-up research question identification exercises are (a) whether or not the questions adequately reflect the priorities of a broad set of stakeholders and (b) whether or not the answers to these questions will be relevant to policymakers and practitioners. We will seek to address both issues in our follow-up work, described below. With regard to the former, there is always the potential for the final list of important questions to reflect the personal perspectives of workshop participants rather than of the stakeholder community as a whole. However, the methodology outlined by Sutherland et al. (2011), and which we followed for this GCRR project, has been used extensively in different domains and has proved to be robust providing there is sufficient input during the question solicitation phase and a strategic selection of workshop participants who represent a diverse view of sectors and roles. For the GCRR project we feel these criteria have been adequately met to generate a robust initial list of priority questions that represent diverse stakeholders. In follow-up surveys to prior question identification exercises, respondents who were not part of the original research question identification process were asked to suggest replacement research questions for those in the existing list which they had ranked as lowest priority: results from those surveys suggested that gaps in research topics were limited (Rudd and Fleishman 2014; Rudd et al. 2014).

Another important consideration for the GCRR project in particular is whether or not it is generalizable beyond Georgia. The process itself is certainly transferable; it would be straightforward to apply exactly the same methodology to conduct a similar exercise in another state, for example. With regard to the questions themselves, it is difficult to speculate about their transferability to other locations. With a few exceptions (e.g. the coastal questions), most of these questions could certainly be applied to other states. What is less clear, however, is how much priority key stakeholders would place on these questions versus others. This determination is based on a range of locally-relevant factors including the social, economic, or environmental priorities of stakeholders in that state and the extent to which stakeholders feel a question has been sufficiently answered in the context of that state.

## Conclusions and next steps

Through a broad, multi-stakeholder process, we have identified 40 important research questions that, if answered, could lay the groundwork for effective, science-based climate action in Georgia. This list of questions is best thought of as a starting point for further discussion of priorities, research in the state, and deliberation regarding policy priorities (Sutherland et al. 2011; Rudd 2011). To facilitate this further work and address the considerations outlined above, we intend to carry out several next steps to advance the GCRR.

First, in Spring 2018 we will conduct a survey to determine how professionals from Georgia rank the relative importance of the research questions we identified and to provide an opportunity for input on topics we may have missed. This will help us broaden inclusion in the question identification and prioritization process, thereby improving the robustness and policy relevance of the results (Rudd 2011). Second, to further engage policy-makers in Georgia, we intend to conduct follow-up interviews with decision-makers in private, public, and non-profit sectors to increase our understanding of how financial and political considerations may constrain or catalyze particular research in Georgia. Third, we will work with partners in the Georgia Climate Project to explore strategies for public dissemination of policy-relevant information related to these questions.

## Supplementary information

Supplementary information
