# Supplementary material for: Climate research priorities for policy-makers, practitioners, and scientists in Georgia, USA
Source: Environ Manage. 2018 May 23;62(2):190–209. doi: 10.1007/s00267-018-1051-4 (PMC6060861; doi:10.1007/s00267-018-1051-4)
Supplement: Supplementary file 1 — Supplementary information [file 267_2018_1051_MOESM1_ESM.doc]

S1. List of workshop participants, including the sectors, regions, career stage and areas of expertise they represent.

| **Participant** | **Sector** | **Region** | **Area of training/expertise** | **Career Stage** |
| --- | --- | --- | --- | --- |
| **Jessica Abbinett** | Academic | Atlanta area | Health | Student |
| **Merryl Alber** | Academic | Coast | Environmental science | Established |
| **Jesse E. Bell** | Academic | Atlanta area | Environmental science | Established |
| **Cyrus Bhedwar** | NGO | Atlanta area | Energy | Established |
| **Lisa Bianchi-Fossatti** | NGO | Atlanta area | Energy | Established |
| **Marilyn A. Brown** | Academic | Atlanta area | Energy | Established |
| **Kim M. Cobb** | Academic | Atlanta area | Climatology | Established |
| **Juliet Cohen** | NGO | Atlanta area | Law | Established |
| **Matt Cox** | Industry | Atlanta area | Energy | Early career |
| **David D'Onofrio** | Government | Atlanta area | Transportation | Early career |
| **Myriam Dormer** | NGO | Atlanta area | Urban sustainability | Established |
| **Nyasha Dunkley** | Government | Atlanta area | Meteorology and climatology | Established |
| **Carrie Furman** | Academic | Middle Georgia | Social science | Early career |
| **Jill Gambill** | Academic | Coast | Societal adaptation | Early career |
| **Jairo Garcia** | Government | Atlanta area | Urban sustainability | Established |
| **Mindy Goldstein** | Academic | Atlanta area | Law | Established |
| **Garry Harris** | NGO | Atlanta area | Energy | Established |
| **Melissa Hopkinson** | Academic | North Georgia | Climatology | Early career |
| **Jean-Ann James** | Foundation | Atlanta area | Energy | Early career |
| **Susan Kidd** | Academic | Atlanta area | Energy | Established |
| **Pam Knox** | Academic | Athens | Agriculture / climatology | Established |
| **John Lanier** | Foundation | Atlanta area | Law | Established |
| **Yang Liu** | Academic | Atlanta area | Health | Established |
| **Daniel C. Matisoff** | Academic | Atlanta area | Energy | Established |
| **Michael D. Meyer** | Government | Atlanta area | Transportation | Established |
| **Jamie Mitchem** | Academic | North Georgia | Climatology | Established |
| **Althea F. P. Moore** | Academic | Coast | Environmental science | Early career |
| **Katherine Moore** | NGO | Atlanta area | Urban sustainability | Established |
| **Stephen O'Day** | Law | Atlanta area | Law | Established |
| **Aspen J. Ono** | Academic | Atlanta area | Policy | Student |
| **Katie Ottenweller** | Law | Atlanta area | Law | Established |
| **Jon Philipsborn** | Industry | Atlanta area | Mitigation/societal adaptation | Established |
| **Mark Risse** | Academic | Coast | Environmental science | Established |
| **Daniel Rochberg** | Academic | Atlanta area | Policy | Established |
| **Murray Rudd** | Academic | Atlanta area | Social science and economics | Established |
| **Shubhayu Saha** | Government | Atlanta area | Health | Established |
| **Paul J. Schramm** | Government | Atlanta area | Health | Early career |
| **Kerrie M. Sendall** | Academic | Middle Georgia | Environmental science | Early career |
| **Fatemeh Shafiei** | Academic | Atlanta area | Social Science | Established |
| **Marshall Shepherd** | Academic | Athens | Meteorology and climatology | Established |
| **Venessa Sims** | Government | Atlanta area | Societal adaptation | Established |
| **Julia Teebken** | Academic | Atlanta area | Social science | Student |
| **Ashby Worley** | NGO | Coast | Environmental science | Early career |

S2. Full list of submitted questions from online solicitation, organized by topic based on the workshop break-out sessions where each was discussed. We have also included the dropped questions, which were not included for consideration at the workshop because they did not fit within the scope of the exercise or duplicated other submitted questions. All the questions are presented here verbatim from the original submissions.

# Agriculture and Natural Resources

1. What policies could help encourage carbon sequestration practices in rural Georgia?

2. Georgia’s dollar agricultural industry is built on years of climate data that is arguably less reliable given climate change. Do we have a plan to transition given the climate will likely behave unpredictably and hotter for longer? If so, would we be able to replace the current industry totals?

3. What public support can be provided to the agricultural sector to reduce methane emissions, particularly in livestock production?

4. What types of information, tools, delivery systems (e.g., web or smartphone applications, email, SMS email alerts) would best serve the agriculture and forest landowners and managers across Georgia.

5. What are the economic benefits if any of climate change in GA (i.e. would changing growing seasons benefit GA agriculture)?

6. What type of impact could Georgia have on carbon sequestration and combating climate change if half of Georgia’s agricultural crop land was in organic production?

7. How much of Georgia's agricultural sector would be impacted if climate change led to increased drought in the state?

8. What can farmers in Georgia do to build resilience on their farms to better handle extreme weather events that are a result of climate change?

9. Is there any mapping of food supply systems for Georgia and how climate change will impact it?

10. What crop and garden plant options are out there or in development that can Keep GA AG in the forefront of profitability and resilience

11. How does agriculture in Georgia best aid in carbon-neutrality for the state, and what are the costs and benefits?

12. How much is climate change projected to cost the primary sectors of the agriculture industry in Georgia over the next 20, 50, 100 years?

13. What pest species such as Johnson grass (Sorghum halepense) are becoming problems for farmers in Georgia as a result of increased average temperatures?

14. What are the top 5 innovative farming techniques that reduce impact on the environment and can help Georgia farmers adapt to a changing climate?

# Weather and Hazards

15. Are there particularly climate-vulnerable populations and regions in the state of Georgia?

16. Can we determine the attribution of climate change on extreme events currently affecting Georgia?

17. Some evidence suggests average air temperature lows are rising at a faster rate than average highs? Why, and what are the potential consequences, on human and natural communities?

18. Is precipitation becoming more variable? Across what timescale?

19. Are extreme events, especially regional droughts and tropical storms, becoming more common or less common? More severe or less severe? Affecting parts of the state differently than in the past?

20. What are the real historical trends of changing temperatures, weather patterns, and sea level?

21. How will climate change shift natural disaster threats in GA and our emergency preparedness in response to potential new threats?

22. If you are over 30 or 40 or 50 how many catastrophe classified as 1% chance(i.e.flooding)are more frequent. Some events occur 3 times/year now but,my mother use to see such events over a 10year period, my grandmother 30-40 years.

23. What are the short term, medium term and long term economic cost/risks of climate change and extreme weather?

24. Is Georgia's Hazard Mitigation Strategy a potential ally to address climate change related challenges or will the issue as such be co-opted by being addressed through a disaster risk reduction framework?

25. Will climate change increase the frequency or severity of weather extremes in Georgia (e.g., tornadoes, hurricanes, ice storms, hail, damaging winds)?

26. Are heatwaves expected to increase in frequency and/or severity in Georgia, and do we have the awareness and resources to deal with them?

27. What climate change impacts, including extreme weather events, have on Georgia's economy? And individual economies including agriculture? Other businesses that depend on the environment and natural resources?

28. What are the affects of climate change on Georgia's natural resources? This includes the direct affects of drought, increased temperature, catastrophic weather events, etc and indirect affects such as increase in wildland fire risk and the risks of non-native invasives, exotics.

29. What are the predicted future precipitation patterns for the southeast and how can communities best prepare for droughts, floods, fire and more extreme weather events in terms of sustainable water supplies and reducing flood hazards and fire management?

# Energy and Mitigation

30. What is the cost of electrification of the vehicle fleet, particularly passenger cars?

31. Adapting the Utility Business Model-- Are utility rate structures designed effectively to manage climate risks?

32. Energy and carbon abatement wedges and supply curves in the Southeast—How big are they?

33. Carbon trading options for the Southeast—How can they be designed?

34. The impact of large-scale EV penetration—How much CO2 mitigation could result?

35. What are the most effective options for reducing the emission of greenhouse gases within the authority and activities of state and local government in Georgia?

36. How can increasing the percentage of EE and DER in the state's energy portfolio reduce reliance on carbon emitting power plants?

37. How can policy be used to offset some of the major sources of carbon emissions in Georgia, such as from vehicles?

38. What are the detailed economic benefits to moving toward energy efficiency and renewables in terms of speed to address energy needs and conversion, jobs created and money kept in the state, regardless of whether one politicizes climate change or not?

39. How would Georgians fare with a revenue-neutral carbon tax and dividend approach (like that advocated by the Climate Leadership Council)?

40. What existing strengths can Georgia leverage to make the conversion to carbon-neutral by 2030? What are the best examples in the state to date that have already started this process?

41. What is the carbon budget for the state of Georgia, and where are the best leverage points for reducing greenhouse gas emissions quickly?

42. Can we transition Georgia's energy system to be 100% renewable energy? Over what time frame would that be practical? Can we do it without including nuclear energy? What is the cost increase or decrease of doing this? Will the energy be reliable at all times?

43. How can we make renewable energy such as solar a part of the state grid faster and more affordably?

44. What policies and structures need to be created to address the intermittency of renewable energy supplies, such as solar and wind?

45. What is the potential for rail electrification in Georgia -- esp. re: goods transport from the Port of Savannah?

# Ecological Systems

46. What species of native plants and animals are most at risk of population declines or local extirpation due to climate stresses? How do climate stresses interact and compare with other environmental stressors on plants, fish, and wildlife?

47. Identifying coastal resilience mitigation of associated vulnerabilities of endemic species & at-risk populations in storm surge coastal communities.

48. How easily can flora and fauna adapt to changing climate at the rate of change we are now experiencing?

49. How does climate change affect Long Leaf Pine coming out of dormancy (candling) and how does that affect our burning?

50. Wire grass. Is the season to burn it to get good reseeding results changing. Is wire grass temperature dependent or day/night length dependent?

51. Are there measurable effects of changes in climate parameters on Georgia’s natural communities or native species? If so, what are they, and which species and/or communities? Do impacts vary by region?

52. Is there any evidence for GA native species altering their latitudinal or elevational distributions consistent with changing climate parameters? Is there any evidence for Florida native species migrating into GA? If so, what species, in what habitats/communities/ecosystems?

53. How climate change will affect the paths of migratory birds in Georgia?

54. Will warming temperatures facilitate the spread of subtropical species, including invasive species and pathogens, into south Georgia?

55. How will climate change affect biodiversity in Georgia, including plant and animal species endemic to the state?

56. What sorts of changes are anticipated in marshland ecosystems in Coastal Georgia due to climate change? And how do we prioritize the needs of marshlands adaptation to sea level rise?

57. What is the impact of the discharge of heated water from electric utility generating plants? What is the impact of cooling towers from the same? These may net-out each other perhaps?

58. What marine species, which are unique to Coastal Georgia (e.g., endangered, commercial value, cultural value) are most vulnerable to climate change?

59. How will sea level rise and flooding impact coastal species, habitats, and ecosystem services?

# Education & Perception

60. What databases are available or need to be better developed to provide relevant, forecasting information to shareholders at the appropriate scale and time-frame

61. Education in which aspects or impacts (human health, air quality, water quality, economic, social, etc.) of climate will allow the scientific community to better reach the general public?

62. What are the political, economic, physical, cultural, technological, or social barriers to adopting solar energy (utility scale and/or rooftop) in the state of Georgia? Can those barriers be addressed or removed to reduce our dependence on coal and natural gas from other states and countries?

63. What percentage of Georgia's residents see climate change as a real threat to society, and where are the major disconnects for those who do not believe it to be a real threat?

64. In rural Georgia, what barriers exist to understanding climate change and/or impacts of climate change?

65. How can we change Georgia policy-maker opinions about climate change? e.g., what's the best way to frame and present climate change information so that it is salient and convinces them of the need for action

66. How can we promote scientific literacy about climate change among the general public, and scientifically sound public policy among decision makers, particularly in coastal communities threatened by sea level rise?

67. How should the research, outreach and extension communities frame the challenges and opportunities related to climate change, including messages around forecasting, uncertainty, economics and possible responses?

68. How can the media effectively communicate climate change to Georgia citizens in a way that bridges partisan lines and values?

69. How do scientists effectively communicate the science and projected impacts of climate change to the public, the media, politicians, teachers, and students in Georgia?

70. What are the climate change attitudes of Georgians in different areas of the state? Is there support for climate change policy in the state?

71. What is the climate literacy of Georgians in different regions of the state?

72. Which of typical human activities contributes the most to emissions? (i.e. vehicles, air conditioning, etc) Which type of power generation produces the fewest emissions/environmental concerns? (nuclear, wind, coal, etc)

73. Is there a way to calculate the total cost of "adaptation"? And once we incur that, how long will each adaptation last, if climate continues to change? Or change accelerates?

# Human Health Linkages

74. What is the health/exposure effect of nuisance tidal flooding and/or storm surge in coastal Georgia?

75. What is the total direct and indirect economic cost to Georgia's economy of both Mercury and Particulate pollution due to coal and natural gas combustion, including human sickness, mortality and reduced or lost economic productivity.

76. What are the short term and long term implications of climate change to our individual health and the health of our communities. Specifically, how can changing environments contribute to asthma, cancer rates, etc.?

77. What diseases, whether insect-borne or not, would be most difficult to manage in Georgia's climate if it were to change in accordance with moderate climate projections?

78. What are the economic impacts of the negative human health effects of climate change?

79. Are there measurable effects (e.g., on health, daily activities, etc.) on human populations of changes in climate parameters? If so, what are they, and which communities? Do the effects vary by region?

80. What are the health effects of pollution and climate change we are seeing today? Particularly for the sensitive populations, like the youth and the elderly?

81. Is there any mapping describing equity, health an climate change impacts in Georgia?

82. As Georgia's climate becomes more tropical are new epidemics possible or likely? what will the impact be on vulnerable populations (infants, children and seniors)?

83. What are the most likely health impacts from climate change in Georgia? What is the distribution of the population vulnerable to these health risks? What are the current and potential actions that can protect these vulnerable populations from climate change?

84. As subtropical and tropical species of plants, animals, viruses, and illnesses move poleward what threats does Georgia face, and are we prepared to cope with them?

85. In terms of urban gardens and restoration, are we planting long-lived species (e.g., longleaf pine) that will be well-adapted to the climate in 50 or 100 years?

# Water-Related Issues

86. How might predictions about future precipitation patterns affect how we prioritize aquatic connectivity projects (aging dams and undersized culverts) and fire management/restoration opportunities compared with current prioritization (e.g., SEACAP, LANDFIRE)?

87. How do changing weather patterns, especially flooding rainfall, affect water quality with regards to enteric pathogens? Specifically, what are the impacts to water used for drinking (i.e., groundwater (well water)) or water used for recreation?

88. Are average water temperatures in rivers and streams, and in coastal waters increasing? At what rate, and across what timescale? With what effects on native flora & fauna?

89. Is the hydrology of major rivers in GA changing as a consequence of changing precipitation/drought/ tropical storm patterns? With what effects on human uses of the water? With what effects on native species?

90. Do we need to build more reservoirs to store more water if rainfall is reduced?

91. Would the systematic opening of storm water systems to ensure that water flows that are currently piped in urban environments contribute positively to climate change?

92. Seemingly many areas of the US are facing extreme drought followed by extreme flooding events. Do we need more water storage for drought and stormwater capture for flooding as a nation?

93. Will Georgia be faced with more frequent and/or severe drought (or flood) conditions in a warmer world? Will our water resources keep up with demand?

94. What saltwater intrusion effects in coastal rivers can be expected under different climate change scenarios?

95. What are the most important aquatic/water-related issues for GA to address assuming that future precipitation patterns will likely include more frequent and more severe periods of drought followed by extreme precipitation events?

96. What are the expected impacts of sea level rise on potable drinking water (both surface & groundwater) in coastal communities?

97. What are the likely impacts of climate change on regional rainfall, and water-flow in the Chattahoochee River?

98. I'd love to see more research done on climate change, increased flooding and the impact this has on human lives, property damage, and changing property values.

# Urban and Regional Issues

99. How is urbanization (in combination with greenhouse-related warming) affecting the climate system?

100. What is the best way for Georgia communities to reach net zero energy use? This would be a great topic for a research experiment

101. How is Georgia prepared to deal with resilience and reliability of its infrastructure when dealing with major climate disasters?

102. Can low income communities save money and increase resiliency through energy efficiency and clean energy access?

103. What will be the implications of climate change related to migration patterns in Georgia, specifically for Atlanta?

104. How can pedestrian deal with extreme heat?

105. To what extent does human climate adaptation play a role in Atlanta's Climate Action Plan? What concerns related to human vulnerability are part of Atlanta's climate and resilience planning?

106. How is urbanization, increased rainfall intensity, and assumptions of stationarity in engineering design affecting urban flooding in cities like Atlanta?

107. How will Georgia regulate driverless vehicles, particularly during a possible era when both driverless and people driven vehicles are on the roads together? Will driverless electric vehicles improve safety and environmental conditions?

108. To what extent are the different departments of Atlanta interlinking in order to address climate change related challenges together?

109. How can reducing household energy burdens through energy efficiency make families more resilient to extreme hot or cold weather conditions?

110. How will climate change impact property insurance?

111. Would Urban Development Policies that ensure that a highly robust and contiguous urban canopy is created while revitalizing old developments and building new developments have a positive impact on climate change?

112. How can Georgia provide incentives to improve mass transit in the region to reduce emissions and create a faster, safer, cleaner transportation system? Does that include MARTA, fast trains, subway, electric vehicles, bike lanes, walking trails, more airports, buses, other forms of transportation?

113. How can increasing access among low income households to community solar improve the resiliency of these households during extreme weather?

# Coastal and Marine Issues

114. What impact will changes in the ocean (temperature increase, acidification, reduced oxygen levels, sea level rise, ocean circulation) have on Georgia's climate, coasts, and economy?

115. What are current patterns of investment in coastal communities, especially in the residential real estate sector, and what are the effects of these patterns on local communities and ecosystems? How can different disciplinary perspectives help us understand these changing patterns and their effects?

116. How will salt marshes adapt to a rising sea level? What will the production changes in the salt marsh change estuary dynamics?

117. How will sea level rise impact the economy of Georgia, particularly the port of Savannah, tourism along the coast, coastal infrastructure, and real estate prices?

118. What are the impacts of climate change on sea farms in Coastal Georgia? And in the contrary can seafarms play a role in climate change adaptation?

119. How would the extent of the dead zone change in the Georgia Coast under climate change?

120. What is the rate of ocean shoreline erosion by specific location over time? Suggest using a combination of remote imagery and on-the-ground data collection and monitoring devices.

121. What are the predictions and potential impacts of combined coastal and riverine flooding with sea level rise?

122. Is the rate of sea level rise increasing along the GA coast? Is the rate of change the same or different along different portions of the coast (within and beyond GA)? If it’s changing at different rates in different regions, why?

123. What economic value (natural capital value) can be ascribed to Georgia's coastal wetlands -- e.g., hurricane protection, storm surge buffering, etc.?

124. What is the influence of increased water temperature on the prevalence and severity of black-gill disease on commercial shrimp landings?

125. What are the impacts of climate change on fisheries in Coastal Georgia?

126. How will climate change impact access to food and water in Coastal Georgia?

127. What local industries are threatened by climate change in Coastal Georgia?

# Dropped questions: These questions are either inconsistent with the scope of the exercise or duplicative of others that were included.

128. Can Al Gore be hired by the State to reverse Climate Change?

129. Climate change will take place and not be altered by human activities; so do nothing.

130. We need to better define what is Climate Change and specifically what is the man made part of this? If this is not defined and agreed upon in a non-politically biased manner, then you will never get the majority of the public on board with this.

131. What are the health impacts associated with climate change in Georgia?

132. How would climate change affect Georgia's agriculture?

133. How Climate Change will affect agriculture in Georgia?

134. Please tell the best strategies to adapt to inevitable climate change.

135. How often is the causeway flooding to Tybee Island per year compared to previous years?

136. How many temperatures annually are being broken across the state?

137. Are average air temperatures rising? Are they rising at different rates in different regions of the state?

138. Is the variability of air temperatures increasing? Across what time scale (days, months or years)?

139. Is average monthly or annual precipitation declining? Differently in different regions?

140. How often are tmperature records broken over the last 10 summers and winters?

141. What is the net cost to the Gross State Product of imported fuel? Coal, natural gas, uranium, oil.

142. Does Atlanta's climate adaptation approach fall into the category of reactive or transformative/anticipatory adaptation responses?

143. I'd like to see a forecasted scenario of where we would be in terms of climate change if policies weren't changed to limit emissions.

144. Why should Georgia spend time and tax dollars studying climate science for Georgia when these activities have already been performed by researchers in the past?

145. What other actors within the state of Georgia are active in the field of climate adaptation/mitigation? What actors need to be and can be mobilized?

146. Poison ivy (Toxicodendron radicans) is definitely increasing in Georgia. I have heard that the spread is due to an adaptation to warmer temperatures. This should be investigated.

147. Do different species or subspecies of pitcher plants have different dietary preferences or needs, predation, and is that changing?

148. Henslow sparrows, DNR surveys some transepts at Moody for population studies. if the climate warms will they migrate earlier, have better reproductive results, or possibly not?

149. Pitcher plants. I went out to look at two bogs. One is under the power lines and it had been frozen off in the recent cold spell. The other is under the canopy and it is doing just fine. What effects will climate change have on them

150. What are the expected impacts of increased frequency of nuisance tidal flooding on coastal native species and natural communities?

151. How Climate Change will affect species in Georgia?

152. Based on best available information, what will life in GA be like in fifty years.

153. What are the best examples of how states and countries have begun to address the issue of climate change?

154. In the absence of a state wide climate change plan of Georgia and with a climate-skeptic senator and governor, what other means are available to address climate change on a state-wide level?

155. What is the most concise and compelling evidence available to help naysayers understand the issue?

156. If Kyoto or Paris accords only might possibly change global Temperature by a fraction of a Degree in 100 years, why shouldn't we look for other more effective and logical Stratagies?

157. How does man-made pollution and man-made negative environmental impacts in Georgia affect climate? Are we prepared to address Clean Air and Clean Water?

158. What is a possible roadmap for the State of Georgia to get to carbon-neutral using existing technologies by 2030?

159. What are the top 5 solutions for mitigating climate changing emissions from activities in Georgia?

160. How Climate Change will affect watersheds in Georgia?

161. Will climate change adversely affect the Metro's water supply?

162. How will climate change effect the City of Atlanta's new food forest park?

163. How Climate Change will affect the displacement of populations to Atlanta?

164. What development is best adjacent to 5 Points MARTA?

165. Will the population in the Southeast be displaced away from the coast and extremely hot regions toward the Appalachians to escape heat, sea level rise, and mosquito borne illnesses?

166. What are the estimated environmental migration impacts and how do we as a state prepare for them? How do these migration patterns vary from in-state movement vs out-of-state vs international areas?

167. How will sea level rise alter the coast, rivers, and wetlands of Georgia's coast and the species that live there?

168. How sea level rise has the coast of Georgia experienced in the last decade?

169. How widespread are the impacts of nuisance tidal flooding on human communities along the GA coast? How fast is the frequency of impacts increasing?

170. Is there any mapping describing how climate change will affect agriculture in Georgia?

171. What communities are most vulnerable to climate change in the Savannah - Chatham County area? And what indicators establish this?

172. in the last 50 years what has changed in terms of weather events -storms, flooding and paradoxically drought periods longer than usual? more frequent /less and consequences observed that are more frequent while between 1900-1950 were few and far between?

173. What accounts for the lack of pronounced warming in the Southeast over the past several decades compared to the rest of the globe? Does this relate to the generally lower lack of acceptance of climate change among the public and politicians in the South?

174. How many semi-truck trips have increased along Georgia highways?

175. How much carbon has been reduced from total installed solar in the state of Georgia?

176. What effect will a steadily rising fee on carbon have on reducing emissions?

177. What is the total cost of a transition to 100% renewable energy?

178. What is the renewable energy potential of Georgia and how much would that cost?

179. (How) does the office of sustainability collaborate with the different counties of the metro Atlanta regions? What other actors aside from the Mayor's Offices of Sustainability & Resilience are considered vitally important for the governance of climate change?

180. What is the impact of a 1-2 foot rise in sea levels along Georgia's coast? Consider urban and rural impacts, costs of relocation, etc.
